# Neuropeptide Y Promoter Variant rs16147 (-399 T/C) Is Associated With Susceptibility to Polycystic Ovary Syndrome

**DOI:** 10.7759/cureus.104961

**Published:** 2026-03-10

**Authors:** Fulya Yukcu, Raziye Akcilar, Ismail Biyik, Halil Ibrahim Sisman

**Affiliations:** 1 Department of Biophysics, Faculty of Medicine, Kutahya Health Sciences University, Kutahya, TUR; 2 Department of Physiology, Faculty of Medicine, Kutahya Health Sciences University, Kutahya, TUR; 3 Department of Obstetrics and Gynecology, Faculty of Medicine, Kutahya Health Sciences University, Kutahya, TUR; 4 Department of Obstetrics and Gynecology, Kutahya City Hospital, Kutahya, TUR

**Keywords:** genetic susceptibility, neuropeptide y, pcr-rflp, polycystic ovary syndrome, rs16147 promoter variant

## Abstract

Background

Polycystic ovary syndrome (PCOS) is a heterogeneous endocrine disorder with complex genetic and neuroendocrine underpinnings. Neuropeptide Y (*NPY*), a key regulator of energy balance and reproductive function, has been implicated in hypothalamic-pituitary-ovarian (HPO) axis dysregulation. However, data on variants of the *NPY *gene in PCOS remain limited. This study investigated the association between the *NPY *promoter variant rs16147 (-399 T/C) and susceptibility to PCOS.

Methods

In this case-control study, 102 women diagnosed with PCOS according to the Rotterdam criteria and 102 age-matched healthy controls were enrolled. Genomic DNA was extracted from peripheral blood samples, and genotyping was performed using polymerase chain reaction-restriction fragment length polymorphism (PCR-RFLP) analysis. Demographic and anthropometric characteristics were recorded, and hormonal and metabolic parameters, including luteinizing hormone (LH), follicle-stimulating hormone (FSH), estradiol, thyroid-stimulating hormone (TSH), total testosterone, fasting glucose, insulin levels, lipid profile components, and homeostasis model assessment of insulin resistance (HOMA-IR), were evaluated. Genotype-phenotype associations were also explored.

Results

Genotype and allele distributions differed significantly between groups. The CT genotype was detected in 84 women with PCOS (82.4%) compared to 24 controls (23.5%) and was associated with increased susceptibility to PCOS (odds ratio {OR} = 15.1; 95% confidence interval {CI}: 7.65-30.0; p < 0.001). In contrast, the CC genotype was more frequent in controls (78, 76.5%) than in the PCOS group (18, 17.6%). The TT genotype was not observed in either group. Similarly, the T allele was more prevalent in women with PCOS (84, 41.2%) than in controls (24, 11.8%) (OR = 5.25; 95% CI: 3.15-8.73; p < 0.001). No significant associations were observed between rs16147 genotypes and hormonal or metabolic parameters within the PCOS cohort.

Conclusions

The *NPY* promoter variant rs16147, particularly the CT genotype and T allele, may contribute to genetic susceptibility to PCOS rather than to clinical phenotype severity. However, these findings should be interpreted with caution and require validation in larger, multicenter populations.

## Introduction

Polycystic ovary syndrome (PCOS) is a multifaceted hormonal condition affecting women of reproductive age, typically presenting with irregular or absent ovulation, elevated androgen levels, and the appearance of numerous small follicles within the ovaries [1]. Beyond hormonal dysregulation, individuals with PCOS often exhibit metabolic abnormalities, including insulin resistance, obesity, and dyslipidemia, thereby increasing the long-term risk of cardiovascular and metabolic complications [2]. The heterogeneity of PCOS in both clinical presentation and biochemical profile is attributed to the combined influence of genetic, hormonal, and environmental determinants [3].

Recent research has increasingly emphasized the potential neuroendocrine origins of PCOS, highlighting the role of central regulatory circuits in the hypothalamic-pituitary-ovarian (HPO) axis. Among the numerous neuropeptides implicated in this complex network, Neuropeptide Y (*NPY*) has garnered particular interest due to its critical involvement in appetite regulation, energy homeostasis, reproductive function, and stress responses [4]. *NPY *is widely expressed in the central nervous system, particularly in the hypothalamus, where it modulates gonadotropin-releasing hormone (GnRH) secretion and interacts with gonadal steroid signaling. The disruption of *NPY* signaling pathways may therefore contribute to several hallmark features of PCOS, including anovulation, hyperandrogenism, and metabolic dysfunction.

Given the multifactorial nature of PCOS, numerous studies have explored the contribution of genetic polymorphisms to its pathogenesis. Meta-analyses have identified associations between PCOS and several gene variants, including those in *VDR*, *ADIPOQ*, and *CYP11A*, which are involved in steroidogenesis, insulin signaling, and metabolic regulation. Alterations in these pathways may contribute to hyperandrogenism, insulin resistance, and metabolic disturbances, which are key mechanisms underlying the pathophysiology of PCOS [5,6]. Against this genetic and multifactorial background, neuropeptides have emerged as potential mediators linking neuroendocrine disruption to the clinical and metabolic manifestations of PCOS. Among them, *NPY* has received attention due to its dual role in energy homeostasis and reproductive axis modulation.

Experimental and clinical studies have shown that *NPY* can affect GnRH pulsatility, luteinizing hormone (LH)/follicle-stimulating hormone (FSH) secretion, and ovarian function via its central and peripheral receptors, suggesting its relevance in the pathophysiology of PCOS [7]. Additionally, *NPY *has been shown to reduce granulosa cell apoptosis in PCOS, and its receptor expression appears to be altered under hyperandrogenic conditions [8].

The rs16147 (-399 T/C) promoter variant is located in the promoter region of the *NPY *gene. This functional variant has been suggested to influence gene regulatory mechanisms and *NPY* expression. It has also been associated with metabolic traits such as serum leptin levels and body fat distribution [9]. Despite this growing body of evidence, data on genetic variations in the *NPY* gene, particularly the rs16147 polymorphism, remain limited in PCOS. Promoter polymorphisms such as rs16147 may influence the transcriptional activity of the *NPY *gene, potentially altering neuroendocrine signaling pathways involved in ovarian function and metabolic regulation at the cellular level. The present study investigated the association between the *NPY* promoter variant rs16147 and susceptibility to PCOS.

## Materials and methods

Study design and setting

This case-control study was conducted at the obstetrics and gynecology outpatient clinic and physiology laboratory of the Faculty of Medicine at Kutahya Health Sciences University. Ethical approval was obtained from the Non-Interventional Clinical Research Ethics Committee of Kutahya Health Sciences University (approval number: 2023/11-25; dated 11 October 2023), and the study was conducted in accordance with the Declaration of Helsinki. All women enrolled in the study provided written informed consent before participation.

Patient selection

A total of 102 women aged between 18 and 42 years, who presented to the obstetrics and gynecology outpatient clinic and fulfilled the diagnostic criteria for PCOS, were enrolled in the study. The control group comprised 102 women aged between 18 and 42 years who visited the clinic for routine examinations or family planning, had no systemic diseases, were not on any medication, and did not meet the diagnostic criteria for PCOS.

The diagnosis of PCOS was made according to the Rotterdam criteria, which require the presence of at least two of the following: clinical or biochemical hyperandrogenism, ovulatory dysfunction, and polycystic ovarian morphology, after the exclusion of related disorders [10].

Women with systemic conditions such as diabetes mellitus with vascular complications, chronic hypertension, autoimmune or inflammatory diseases, renal or hepatic dysfunction, cardiovascular disorders, or a history of stroke were excluded from the study. In addition, those using medications that influence insulin or lipid metabolism, including glucocorticoids, oral contraceptives, anti-androgens, or ovulation-inducing drugs, were also not eligible.

Data collection

Demographic and anthropometric data, including age, height, weight, body mass index (BMI), hip and waist circumferences, gravidity, and parity, were recorded for both groups. In the PCOS group, laboratory evaluations at admission included hormonal parameters (LH, FSH, estradiol, thyroid-stimulating hormone {TSH}, and dehydroepiandrosterone sulfate {DHEA-S}), total testosterone, fasting glucose, and insulin levels, as well as lipid profile components (total cholesterol, triglycerides {TG}, low-density lipoprotein {LDL}, and high-density lipoprotein {HDL}). Insulin resistance was estimated using the homeostasis model assessment of insulin resistance (HOMA-IR), calculated as fasting insulin (µIU/mL) × fasting glucose (mg/dL) / 405.

Biochemical measurements

Blood samples were obtained on the second or third day of the menstrual cycle. Following an overnight fast, venous blood was collected between 08:00 and 10:00 a.m. into evacuated serum separator tubes containing clot activator (BD Vacutainer® SST™ Advance, Plymouth, United Kingdom). Within 30 minutes of collection, samples were centrifuged at 3000 revolutions per minute (rpm) for 10 minutes. After equilibration to room temperature, biochemical and hormonal analyses were performed using the Beckman Coulter AU5800 and DxI 800 analyzers (Beckman Coulter, Inc., Brea, CA).

DNA isolation and polymerase chain reaction-restriction fragment length polymorphism (PCR-RFLP) analysis

Peripheral blood was collected from the participants in both groups into ethylenediaminetetraacetic acid (EDTA)-coated tubes (2 mL) and stored at -20°C until DNA isolation. Genomic DNA was extracted using the conventional phenol-chloroform method.

The genotyping of the *NPY* promoter variant rs16147 was performed using the polymerase chain reaction-restriction fragment length polymorphism (PCR-RFLP) method. The following primer sequences were used for amplification: forward primer 5′-TTCCTACTCCGGCACCCAGTGGG-3′ and reverse primer 5′-GGGCTTTTATGGAGCTTCCTCGC-3′. PCR amplification was carried out in a total reaction volume of 25 µL, consisting of 12.5 µL of 2× Taq Master Mix (abm, Canada, Catalog Number G013), 1 µL each of forward and reverse primers (0.5 µM), 2 µL of template DNA (100-500 ng), and sterile deionized water to complete the final volume. The thermal cycling protocol included an initial denaturation at 95°C for five minutes, followed by 39 cycles of denaturation at 95°C for 30 seconds, annealing at 61°C for 30 seconds, and extension at 72°C for 30 seconds, with a final extension step at 72°C for 10 minutes.

The PCR products were digested with AluI restriction enzyme (Thermo Scientific, USA), by incubating at 37°C for 16 hours [11]. The resulting fragments were then separated on a 2% agarose gel alongside a 100 base pair (bp) DNA ladder (abm, Canada, Catalog Number G193) and visualized using ethidium bromide staining. To confirm the reproducibility of the genotyping results, a subset of randomly selected samples was re-genotyped, and identical results were obtained.

**Figure 1:**
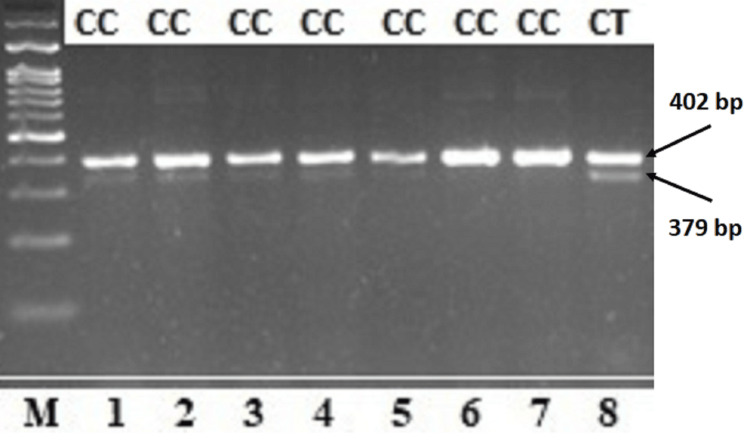
Representative agarose gel electrophoresis image showing PCR-RFLP genotyping of the NPY promoter variant rs16147 (-399 T/C). Lanes 1-7 represent the CC genotype (402 bp), whereas lane 8 represents the CT genotype (402 and 379 bp). The TT genotype (379 bp) was not observed in the study population. M: 100 bp DNA ladder (abm, Catalog Number G193). PCR-RFLP, polymerase chain reaction-restriction fragment length polymorphism; *NPY*, Neuropeptide Y; bp, base pairs

For the genotyping analysis of the *NPY* gene rs16147 polymorphism, genotypes were determined according to the expected fragment sizes after AluI digestion. The amplified PCR product was 402 bp in length. The AluI restriction enzyme recognizes a single restriction site within the amplified fragment. Consequently, the CC genotype yielded an undigested 402 bp fragment, the TT genotype yielded a 379 bp fragment, and the CT genotype produced both 402 bp and 379 bp fragments (Figure 1). However, only the CC and CT genotypes were observed in the study population.

Statistical analysis

Statistical analyses were performed using the SPSS version 27.0 software (IBM Corp., Armonk, NY). The normality of data distribution was assessed using the Kolmogorov-Smirnov test. Normally distributed variables were analyzed using the independent samples t-test, whereas the Mann-Whitney U test was applied for non-normally distributed variables. Continuous variables were expressed as mean ± standard deviation (SD) or median (interquartile range {IQR}), as appropriate. Categorical variables were presented as numbers and percentages. Differences in categorical variables were evaluated using the chi-square (χ²) test or Fisher’s exact test when expected cell counts were <5. Differences in genotype and allele distributions between the PCOS and control groups were evaluated using the χ² test. Odds ratios (OR) with corresponding 95% confidence intervals (CI) were calculated to estimate the association between rs16147 and PCOS.

Sample size determination was based on an a priori power analysis using an OR of 2.195 reported by Katus et al. [12]. Power calculations were performed with the G*Power 3.1 software (Heinrich-Heine-Universität Düsseldorf, Düsseldorf, Germany), assuming a medium effect size (0.5), a one-tailed significance level of 0.05, and a confidence level of 95%. Under these assumptions, a minimum sample size of 102 participants per group was required, yielding an estimated statistical power of approximately 86% to detect differences between two independent groups. A p-value of ≤0.05 was considered statistically significant. Genotype distributions in the control group were also evaluated for compliance with the Hardy-Weinberg equilibrium (HWE) using the χ² test and were found to be in equilibrium (χ² = 1.81; degrees of freedom {df} = 1; p = 0.10).

## Results

Demographic, anthropometric, and clinical characteristics

The median age was comparable between the PCOS and control groups (25.0 versus 26.0 years, p = 0.102). Median weight (55.0 versus 64.0 kg, p < 0.001) and BMI (20.3 versus 23.6 kg/m², p < 0.001) were significantly lower in the PCOS group. Although median height was slightly lower in the PCOS group (163.5 versus 165.0 cm), this difference reached statistical significance (p = 0.05). Waist circumference was significantly higher in women with PCOS (74.0 versus 70.0 cm, p = 0.020), whereas hip circumference was significantly lower (92.0 versus 97.0 cm, p < 0.001).

Reproductive history also showed clear differences. Nulligravidity was more frequent in the PCOS group (84, 82.4%) than in controls (62, 60.8%), and the overall distribution of gravidity differed significantly between groups (χ² = 22.62; df = 3; p < 0.001). Nulliparity was similarly more prevalent in the PCOS group (89, 87.3%) compared to controls (63, 61.8%), with a significant difference in parity distribution (χ² = 20.24; df = 3; p < 0.001). Hirsutism was observed in 46 women with PCOS (45.1%) and in zero controls (0.0%), demonstrating a significant difference between groups (χ² = 59.39; df = 1; p < 0.001). In addition, cigarette smoking was markedly more prevalent in the PCOS group (66, 64.7%) compared to controls (20, 19.6%), with a statistically significant difference (χ² = 42.54; df = 1; p < 0.001).

**Figure 2:**
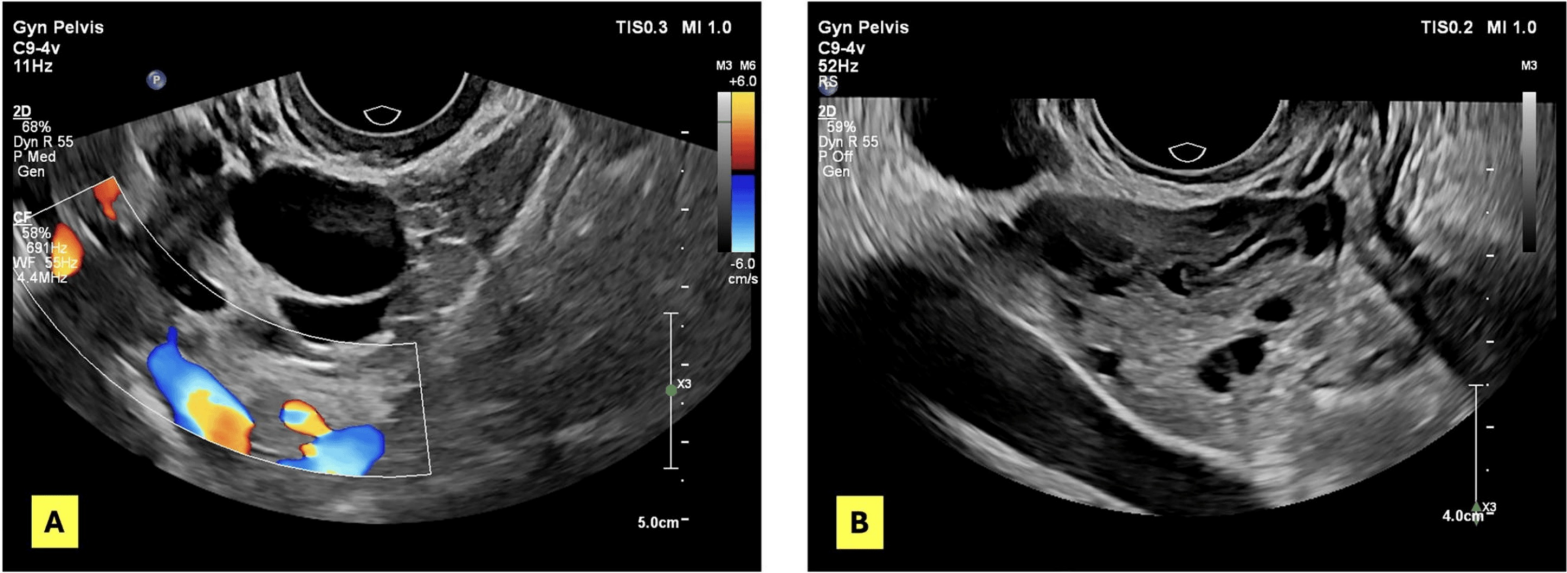
Representative transvaginal ultrasonographic ovarian morphology. Representative transvaginal ultrasonographic images showing a normal ovary in a control subject (A) and polycystic ovarian morphology in a patient with PCOS (B). PCOS: polycystic ovary syndrome

Biochemical analysis showed no significant difference in median FSH, LH, LH/FSH ratio, or TSH levels between the PCOS and control groups (all p > 0.05). Median estradiol levels were significantly lower in the PCOS group compared to controls (37.7 versus 45.2 pg/mL, p = 0.004). Conversely, total testosterone concentrations were markedly higher in women with PCOS (0.62 ± 0.26 ng/dL) than in the control group (0.40 ± 0.22 ng/dL, p < 0.001). These findings reflect the characteristic hyperandrogenic profile observed in women with PCOS (Table 1). Transvaginal ultrasonographic evaluation revealed normal ovarian morphology in the control group, whereas women with PCOS exhibited enlarged ovaries containing multiple peripheral follicles and increased stromal echogenicity, consistent with polycystic ovarian morphology (Figure 2).

**Table 1:** Demographic, anthropometric, and clinical characteristics of women with PCOS and controls. Categorical variables were analyzed using the chi-square (χ²) test or Fisher’s exact test when expected cell counts were <5. *P ≤ 0.05 was considered significant. ^a^Median (IQR: 25-75). ^b^Mean ± standard deviation (SD). IQR, interquartile range; BMI, body mass index; FSH, follicle-stimulating hormone; LH, luteinizing hormone; TSH, thyroid-stimulating hormone; PCOS, polycystic ovary syndrome

Characteristics median (IQR: 25-75)	PCOS (n = 102)	Control (n = 102)	P-values
Age (years)^a^	25.0 (22.0-29.0)	26.0 (24.0-28.2)	0.102
Weight (kg)^a^	55.0 (51.0-59.0)	64.0 (57.7-70.2)	<0.001*
Height (cm)^a^	163.5 (161.0-166.0)	165.0 (162.0-167.0)	0.05*
BMI (kg/m^2^)^a^	20.3 (19.7-21.4)	23.6 (20.7-26.3)	<0.001*
Waist circumference (cm)^a^	74.0 (70.7-78.0)	70.0 (65.0-80.0)	0.020*
Hip circumference (cm)^a^	92.0 (88.0-96.0)	97.0 (93.0-105.0)	<0.001*
Gravidity, n (%)			
0	84 (82.4)	62 (60.8)	<0.001*
1	14 (13.7)	11 (10.8)	
2	3 (2.9)	22 (21.6)	
3	1 (1.0)	7 (6.8)	
Parity, n (%)			
0	89 (87.3)	63 (61.8)	<0.001*
1	9 (8.8)	16 (15.7)	
2	3 (2.9)	21 (20.5)	
3	1 (1.0)	2 (2.0)	
Hirsutism, n (%)			
Yes	46 (45.1)	0 (0.0)	<0.001*
No	56 (54.9)	102 (100.0)	
Cigarette smoking, n (%)			
Yes	66 (64.7)	20 (19.6)	<0.001*
No	36 (35.3)	82 (80.4)	
FSH (mIU/ml)^a^	5.89 (4.44-7.12)	5.67 (4.88-7.01)	0.949
LH (mIU/ml)^a^	7.89 (5.07-9.89)	6.77 (5.05-9.69)	0.257
LH/FSH^a^	1.28 (0.82-1.91)	1.13 (0.88-1.58)	0.224
Estradiol (pg/mL)^a^	37.7 (29.7-51.5)	45.2 (33.6-66.9)	0.004*
TSH (mIU/mL)^a^	1.70 (1.23-2.07)	1.53 (1.18-2.08)	0.466
Total testosterone (ng/dL)^b^	0.62 ± 0.26	0.40 ± 0.22	<0.001*

Genotypic and allelic distribution of the *NPY* promoter variant rs16147

In the control group, the CC genotype was observed in 78 individuals (76.5%) and the CT genotype in 24 individuals (23.5%). In the PCOS group, 18 individuals (17.6%) had the CC genotype, whereas 84 (82.4%) carried the CT genotype. The TT genotype was not detected in either group (0%). Genotype distribution differed significantly between the two groups (χ² = 70.8; df = 1; p < 0.001). Regarding allele frequencies, the C allele was more common in the control group (180, 88.2%) than in the PCOS group (120, 58.8%), whereas the T allele was more frequent in the PCOS group (84, 41.2%) compared to controls (24, 11.8%). This difference in allele distribution was also statistically significant (χ² = 45.3; df = 1; p < 0.001) (Table 2).

**Table 2:** Genotypic and allelic distribution of the NPY promoter variant rs16147 (-399 T/C) between women with PCOS and controls. *P ≤ 0.05 was considered statistically significant. *NPY*, Neuropeptide Y; PCOS, polycystic ovary syndrome; OR, odds ratio; CI, confidence interval

*NPY* promoter variant rs16147 (-399 T/C)	Control (n = 102)	PCOS (n = 102)	OR (95% CI)	P-values
Genotypes, n (%)				
CC	78 (76.5)	18 (17.6)	1 (reference)	
CT	24 (23.5)	84 (82.4)	15.1 (7.65-30.0)	<0.001*
TT	0 (0.0)	0 (0.0)	-	-
	χ² = 70.8; df = 1; p < 0.001			
Alleles, n (%)				
C	180 (88.2)	120 (58.8)	1 (reference)	
T	24 (11.8)	84 (41.2)	5.25 (3.15-8.73)	<0.001*
	χ² = 45.3; df = 1; p < 0.001			

Genotype-based comparisons within the group

Control Group

In the control group, demographic, anthropometric, and clinical parameters, including age, weight, height, BMI, waist and hip circumference, gravidity, parity, smoking status, and hirsutism, were compared between individuals with CC and CT genotypes of the *NPY* promoter variant rs16147. The median age, BMI, waist, and hip circumference were comparable between the groups (all p > 0.05). Gravidity, parity, and smoking rates did not differ significantly, and no cases of hirsutism were observed in either genotype group. Likewise, hormonal parameters, including FSH, LH, LH/FSH ratio, estradiol, TSH, and total testosterone levels, were similar between genotypes (all p > 0.05) (Table 3).

**Table 3:** Demographic, anthropometric, and clinical characteristics of the control group according to NPY promoter variant rs16147 (-399 T/C) genotypes. P ≤ 0.05 was considered significant. Categorical variables were analyzed using the chi-square (χ²) test or Fisher’s exact test when expected cell counts were <5. ^a^Median (IQR: 25-75) ^b^Mean ± standard deviation (SD). IQR, interquartile range; BMI, body mass index; FSH, follicle-stimulating hormone; LH, luteinizing hormone; TSH, thyroid-stimulating hormone; *NPY*, Neuropeptide Y

Characteristics median (IQR: 25-75)	CC	CT	P-values
Age (years)^a^	26.0 (24.0-29.0)	25.0 (23.0-28.0)	0.223
Weight (kg)^a^	64.0 (57.7-71.0)	63.5 (56.0-69.7)	0.743
Height (cm)^a^	165.0 (160.7-167.0)	165.0 (162.2-166.7)	0.736
BMI (kg/m^2^)^a^	23.7 (20.7-26.6)	23.1 (20.3-25.6)	0.554
Waist circumference (cm)^a^	70.5 (65.0-80.0)	69.5 (65.0-75.7)	0.564
Hip circumference (cm)^a^	97.5 (93.0-105.0)	96.5 (93.2-106.0)	0.816
Gravidity, n (%)			
0	50 (64.1)	12 (50.0)	0.387
1	8 (10.3)	3 (12.5)	
2	14 (17.9)	8 (33.3)	
3	6 (7.7)	1 (4.2)	
Parity, n (%)			
0	50 (64.1)	13 (54.1)	0.562
1	12 (15.4)	4 (16.7)	
2	14 (17.9)	7 (29.2)	
3	2 (2.6)	0 (0.0)	
Hirsutism, n (%)			
Yes	0 (0.0)	0 (0.0)	-
No	78 (100.0)	24 (100.0)	
Cigarette smoking, n (%)			
Yes	17 (21.8)	3 (12.5)	0.316
No	61 (78.2)	21 (87.5)	
FSH (mIU/mL)^a^	5.89 (4.35-7.47)	5.89 (4.47-7.05)	0.500
LH (mIU/mL)^a^	8.30 (6.10-9.83)	7.74 (4.90-9.90)	0.931
LH/FSH^a^	1.52 (1.03-1.91)	1.27 (0.81-1.93)	0.290
Estradiol (pg/mL)^a^	38.4 (29.1-51.3)	37.0 (29.6-52.5)	0.096
TSH (mIU/mL)^a^	1.46 (1.04-2.02)	1.73 (1.26-2.08)	0.194
Total testosterone (ng/dL)^b^	0.38 ± 0.21	0.47 ± 0.22	0.092

PCOS Group

In the PCOS group, comparisons of demographic, anthropometric, and clinical parameters between CC (n = 18) and CT (n = 84) genotypes of the NPY promoter variant rs16147 revealed no statistically significant differences. The median age was slightly higher in the CC genotype group compared to the CT group (28.0 versus 24.0 years, p = 0.226). Weight, height, and BMI values were also similar between the two groups (all p > 0.05). Waist and hip circumferences showed comparable values across genotypes (75.0 versus 73.0 cm, p = 0.474, and 94.0 versus 92.0 cm, p = 0.244, respectively). Reproductive history parameters were similarly distributed. Nulligravidity was observed in 13 women with the CC genotype (72.2%) and 71 women with the CT genotype (84.5%) (p = 0.543). Nulliparity was present in 15 CC carriers (83.3%) and 74 CT carriers (88.1%) (p = 0.828). Hirsutism was observed in six women with the CC genotype (33.3%) and 40 with the CT genotype (47.6%), without statistical significance (p = 0.269). Cigarette smoking was reported in 13 CC carriers (72.2%) and 53 CT carriers (63.1%) (p = 0.462). The evaluation of hormonal markers, including FSH, LH, estradiol, TSH, total testosterone, and DHEA-S, demonstrated comparable values across genotypes (all p > 0.05). Similarly, metabolic parameters such as lipid profile components, fasting glucose, insulin, and HOMA-IR did not significantly differ between the groups (Table 4).

**Table 4:** Clinical, hormonal, and metabolic characteristics of the PCOS group according to genotypes of the NPY promoter variant rs16147. P ≤ 0.05 was considered significant. Categorical variables were analyzed using the chi-square (χ²) test or Fisher’s exact test when expected cell counts were <5. ^a^Median (IQR: 25-75). ^b^Mean ± standard deviation (SD). IQR, interquartile range; BMI, body mass index; FSH, follicle-stimulating hormone; LH, luteinizing hormone; TSH, thyroid-stimulating hormone; DHEA-S, dehydroepiandrosterone sulfate; TG, triglyceride; HDL, high-density lipoprotein; LDL, low-density lipoprotein; HOMA-IR, homeostasis model assessment of insulin resistance; PCOS, polycystic ovary syndrome; *NPY*, Neuropeptide Y

Characteristics median (IQR: 25-75)	CC	CT	P-values
Age (years)	28.0 (22.5-31.0)	24.0 (22.0-28.0)	0.226
Weight (kg)	56.0 (52.0-60.2)	55.0 (51.0-58.0)	0.538
Height (cm)	163.0 (159.0-167.0)	164.0 (161.5-166.0)	0.898
BMI (kg/m^2^)	20.5 (20.0-21.8)	20.3 (19.7-21.3)	0.527
Waist circumference (cm)	75.0 (71.7-79.5)	73.0 (70.0-78.0)	0.474
Hip circumference (cm)	94.0 (89.7-98.0)	92.0 (88.0-95.0)	0.244
Gravidity, n (%)			
0	13 (72.2)	71 (84.5)	0.543
1	4 (22.2)	10 (11.9)	
2	1 (5.6)	2 (2.4)	
3	0 (0.0)	1 (1.2)	
Parity, n (%)			
0	15 (83.3)	74 (88.1)	0.828
1	2 (11.1)	7 (8.3)	
2	1 (5.6)	2 (2.4)	
3	0 (0.0)	1 (1.2)	
Hirsutism, n (%)			
Yes	6 (33.3)	40 (47.6)	0.269
No	12 (66.7)	44 (52.4)	
Cigarette smoking, n (%)			
Yes	13 (72.2)	53 (63.1)	0.462
No	5 (27.8)	31 (36.9)	
FSH (mIU/mL)^a^	5.87 (4.15-7.38)	5.89 (4.52-7.07)	0.792
LH (mIU/mL)^a^	8.30 (6.15-9.80)	7.72 (4.90-9.90)	0.548
LH/FSH^a^	1.63 (1.04-1.93)	1.24 (0.80-1.90)	0.257
Estradiol (pg/mL)^a^	39.1 (29.5-58.2)	36.7 (29.5-50.1)	0.885
TSH (mIU/mL)^a^	1.46 (1.07-1.98)	1.74 (1.26-2.09)	0.219
Total testosterone (ng/dL)^b^	0.67 ± 0.27	0.61 ± 0.26	0.282
DHEA-S (µg/mL)^a^	266.5 (208.2-343.5)	252.5 (200.0-305.2)	0.243
Cholesterol (mg/dL)^b^	182.5 ± 30.1	173.9 ± 27.4	0.206
TG (mg/dL)^a^	92.5 (79.0-131.7)	94.0 (68.0-119.2)	0.545
HDL (mg/dL)^b^	55.8 ± 9.67	56.8 ± 11.5	0.618
LDL (mg/dL)^a^	82.5 (76.5-93.7)	98.5 (77.0-112.0)	0.059
Fasting glucose (mg/dL)^a^	89.5 (79.0-98.0)	88.0 (81.2-93.0)	0.550
Fasting insulin (mU/mL)^a^	13.4 (7.64-17.1)	9.65 (7.00-13.0)	0.168
HOMA-IR^a^	2.41 (1.54-3.99)	2.12 (1.46-2.79)	0.226

## Discussion

PCOS is a multifactorial disorder arising from complex interactions among genetic, hormonal, and environmental factors. Although substantial progress has been made in elucidating the metabolic and endocrine abnormalities associated with PCOS, the involvement of neuropeptide signaling pathways remains incompletely understood [13]. *NPY*, a key regulator of appetite, energy homeostasis, and reproductive function, has recently attracted attention as a potential molecular link between metabolic dysregulation and disturbances of the reproductive axis in PCOS. *NPY* may influence the hypothalamic-pituitary-ovarian axis by modulating hypothalamic GnRH secretion and subsequent LH and FSH release [8]. While the *NPY* promoter variant rs16147 has been investigated in various metabolic and neuropsychiatric conditions, evidence regarding its contribution to PCOS susceptibility remains limited. Investigating *NPY* gene variants in this context may provide further insight into the neuroendocrine mechanisms potentially involved in PCOS susceptibility.

In our study, women with PCOS exhibited lower weight and BMI values compared to the control group yet had higher waist circumference measurements. The relatively lower BMI observed in our cohort may reflect sample characteristics and regional factors, as the study population predominantly consisted of young women without major comorbidities. Nevertheless, these findings suggest that central fat accumulation may be evident in PCOS even in the absence of general obesity. Similar “normal weight with increased central adiposity” phenotypes have been described previously. Zhang et al. reported that women with PCOS, including those with normal BMI, showed significantly greater visceral fat accumulation, which was associated with insulin resistance indices such as HOMA-IR and alterations in sex hormone levels [14]. These observations support the concept that central adiposity may represent an important metabolic feature of PCOS rather than solely a consequence of obesity. Likewise, Glintborg et al. demonstrated that women with PCOS had higher central fat deposition, including increased waist circumference and waist-to-hip ratio, compared to controls, highlighting anthropometric parameters as potential metabolic risk indicators even in nonobese individuals [15]. Consistent with previous literature, our findings suggest that central adiposity may represent an important phenotypic characteristic of PCOS even in the absence of obesity, possibly reflecting interactions among genetic, hormonal, and metabolic factors that influence fat distribution.

In addition to anthropometric differences, our study revealed distinct reproductive and clinical characteristics. Nulligravidity and nulliparity were significantly more frequent among women with PCOS (84, 82.4%, versus 62, 60.8%, and 89, 87.3%, versus 63, 61.8%, respectively), consistent with ovulatory dysfunction being a central feature affecting fertility outcomes in this syndrome. These findings are in agreement with the meta-analysis by Bozdag et al., which reported higher infertility rates in PCOS primarily related to ovulatory dysfunction rather than structural abnormalities [16]. Furthermore, hirsutism was observed in 46 women (45.1%) with PCOS, whereas it was absent in the control group, supporting hyperandrogenism as a prominent clinical feature of the syndrome. This prevalence aligns with the wide range (40%-75%) reported in previous large-scale studies, reflecting variability across populations [17,18]. According to Azziz et al., hirsutism affects a substantial proportion of women with PCOS and represents one of the most consistent clinical manifestations of androgen excess [17]. Similarly, Spritzer et al. emphasized that differences in hirsutism prevalence among studies may reflect variations in ethnicity, diagnostic thresholds, and phenotypic heterogeneity within PCOS populations [18]. In addition, cigarette smoking was more prevalent in our PCOS cohort (66, 64.7%) than in controls (20, 19.6%). Previous studies suggest that smoking may exacerbate androgen excess and adversely influence reproductive and metabolic parameters in women with PCOS [19,20]. Collectively, these observations suggest that beyond anthropometric and reproductive characteristics, hyperandrogenism and lifestyle factors such as smoking may contribute to the clinical heterogeneity of PCOS. Given the higher prevalence of smoking in our cohort, lifestyle-related factors may also represent potential environmental influences. However, genotype-based comparisons within both the PCOS and control groups did not reveal smoking-related differences, suggesting that the observed association between the *NPY* promoter variant rs16147 and PCOS is unlikely to be explained solely by lifestyle factors.

In our study, serum estradiol levels were significantly lower in women with PCOS compared to controls, which may reflect hormonal patterns commonly associated with chronic anovulation and follicular arrest in this disorder. Su et al. emphasized that impaired folliculogenesis and disrupted feedback mechanisms may lead to altered estrogen synthesis in PCOS, even in the absence of ovarian failure [13]. Similarly, Ach et al. reported follicular immaturity in high-resolution ultrasonographic evaluations of North African patients with PCOS, accompanied by lower estradiol levels consistent with impaired follicular development [1]. Conversely, total testosterone concentrations were markedly higher in the PCOS group, confirming hyperandrogenism as a hallmark biochemical feature of the syndrome. This observation aligns with findings by Azziz et al., who reported elevated androgen levels in a substantial proportion of women with PCOS [17]. Similarly, Spritzer et al. described hirsutism and biochemical hyperandrogenism as interrelated manifestations of the disorder [18]. No significant differences were observed in FSH, LH, LH/FSH ratio, or TSH levels between the groups. This finding is consistent with previous reports indicating that hormonal patterns in PCOS may vary across phenotypes and are influenced by factors such as ethnicity and body composition [10,16,17].

The present study revealed significant differences in genotype and allele distributions of the *NPY* promoter variant rs16147 between patients with PCOS and controls. The higher frequency of the CT genotype and the markedly increased proportion of the T allele in the PCOS group suggest a potential association of this variant with PCOS susceptibility. However, this association does not imply causality and should be confirmed in larger, independent cohorts. Previous studies have reported that rs16147 may be associated with metabolic and neuropsychiatric outcomes in different disease contexts. For example, Al-Barzanji and Al-Darraji reported a higher frequency of the C allele in individuals with type 2 diabetes, which was associated with metabolic alterations potentially related to changes in *NPY* expression [21]. Similarly, Akel Bilgiç et al. reported that the C allele, particularly in its homozygous form, was more frequent among alcohol-dependent individuals, suggesting a possible genetic contribution to addiction susceptibility [22]. These observations suggest that allele-related associations of rs16147 may vary across disease contexts. In our cohort, the higher frequency of the T allele and CT genotype indicates a potential phenotype-specific association in PCOS, possibly linked to hormonally regulated neuroendocrine mechanisms rather than a uniform effect across conditions. Similarly, the large Estonian birth cohort study by Kiive et al. reported an association between the T allele and CT genotype of rs16147 and psychological traits, particularly among women, supporting the notion that this promoter variant may contribute to sex-related phenotypic variability [23]. Although the TT genotype was not observed in our study, this finding may be explained by the low-frequency homozygous state of the rs16147 variant in the investigated population. The maintenance of the Hardy-Weinberg equilibrium in the control group despite the absence of this genotype supports the reliability of the genotyping results and argues against major population stratification or technical bias. Overall, our findings indicate a potential genetic contribution of rs16147 to PCOS susceptibility; however, replication in larger and ethnically diverse cohorts is warranted. Taken together, these observations suggest that the phenotypic effects of the *NPY* promoter variant rs16147 may vary across metabolic, behavioral, and neuroendocrine contexts.

In the control group, no significant differences were observed between the CC and CT genotypes of the *NPY* promoter variant rs16147 in terms of age, weight, height, BMI, waist and hip circumference, reproductive history, smoking status, or the presence of hirsutism. These findings suggest that in the absence of disease, this genetic variation was not associated with measurable differences in basic clinical characteristics. Similarly, several studies conducted in healthy populations have reported limited genotype-related variability, whereas genotype-phenotype associations appear more frequently under specific pathological conditions [6,7]. This observation supports the notion that the phenotypic impact of rs16147 may be context-dependent and may be influenced by disease-related hormonal or metabolic factors. Nonetheless, the limited number of certain genotypes within the control group reduces statistical power and suggests that subtle effects cannot be entirely excluded.

No statistically significant differences were observed between CC and CT genotypes of the *NPY* promoter variant rs16147 with respect to reproductive history, hormonal markers, or metabolic parameters in women with PCOS. Although the TT genotype was not observed, the maintenance of the Hardy-Weinberg equilibrium in the control group supports the reliability of genotype-based comparisons. This observation suggests that the rs16147 variant was not associated with measurable genotype-based differences in the clinical phenotype of PCOS within our cohort, although indirect effects through broader neuroendocrine or metabolic pathways cannot be excluded. Previous studies have reported that rs16147 is more consistently associated with cardiometabolic traits, such as lipid metabolism, body composition, and leptin levels, rather than with gonadotropin secretion [9,24]. Similarly, while both genotypic groups in our cohort exhibited high rates of nulligravidity and nulliparity, this likely reflects the infertility characteristic of PCOS itself rather than the effect of specific genetic variants, consistent with findings by Hamidi et al. on other polymorphisms related to reproductive outcomes [25]. Furthermore, mechanistic insights by Chen et al. suggest that the inhibitory effects of *NPY* on GnRH secretion may be mediated primarily by receptor-level interactions rather than directly by the rs16147 polymorphism [7]. Mutschler et al. reported that the CT genotype was more frequent among women, whereas carriers of the TT and CC genotypes exhibited higher waist-to-hip ratios compared with CT carriers [9]. In line with this observation, our study also found a higher frequency of the CT genotype among women with PCOS; however, no significant genotype-based differences in anthropometric parameters were detected. Taken together, these findings suggest that potential metabolic associations of rs16147 may vary according to population characteristics, disease status, and environmental influences. While previous reports have linked homozygous genotypes to central obesity, the absence of significant genotype-related differences in our cohort highlights the multifactorial nature of metabolic phenotypes in PCOS.

Moreover, Franzago et al. reported that the *NPY* promoter variant rs16147 may modulate physiological responses to environmental exposures, such as dietary interventions, supporting its potential relevance to complex traits such as PCOS. In their study, carriers of the C allele exhibited an attenuated metabolic response to Mediterranean diet-based lifestyle modifications, suggesting that this promoter variant may influence not only disease susceptibility but also phenotypic characteristics such as body composition and metabolic adaptability [26]. Collectively, these observations suggest that while rs16147 may be linked to susceptibility to PCOS, it was not associated with clear genotype-based differences in reproductive or hormonal parameters within our cohort, indicating that phenotypic effects are likely shaped by environmental and population-specific contexts. Given the pilot nature of this study, the present findings should be interpreted as hypothesis-generating rather than definitive evidence of causality.

Limitations

This study has several limitations that should be acknowledged. Although the sample size was supported by an a priori power analysis, the overall cohort size may have limited the detection of rare homozygous genotypes. The absence of the TT genotype likely reflects the low minor allele frequency of this variant in the investigated population rather than methodological limitations. However, this finding may be related to the low minor allele frequency reported for this variant in some populations. The presence of the HWE in the control group supports the reliability of the genotyping results, although the inclusion of a TT-positive control would further strengthen methodological confirmation. As this investigation was designed as a pilot study focusing on genetic predisposition, the assessments of *NPY* gene expression or circulating serum *NPY* levels were not performed, which could have provided additional mechanistic insight. Moreover, lifestyle and environmental factors such as diet, physical activity, and stress were not systematically evaluated, limiting a comprehensive assessment of gene-environment interactions. The single-center design and recruitment from a specific geographic region may further restrict the generalizability of the findings. Nevertheless, the use of standardized diagnostic criteria, the homogeneity of the study groups, and the application of a robust genotyping methodology strengthen the reliability and internal validity of our results.

## Conclusions

Our findings indicate that the *NPY* promoter variant rs16147 may be associated with susceptibility to PCOS. The higher prevalence of the CT genotype and T allele in the PCOS group, in contrast to the predominance of the CC genotype and C allele in controls, points to a genetic predisposition to the syndrome. The lack of genotype-related differences in hormonal and metabolic parameters indicates that this variant primarily influences disease susceptibility rather than shaping the clinical phenotype. Overall, these results underscore the potential involvement of neuropeptide-related pathways in genetic susceptibility to PCOS, rather than in determining overt clinical or hormonal manifestations. However, since this was designed as a pilot study, our conclusions should be confirmed by larger, multicenter investigations in diverse populations. Future research incorporating functional analyses, specifically *NPY* gene expression and serum protein levels, will be essential to fully clarify the biological impact of the rs16147 polymorphism in PCOS.
